# Identification of scaffold/Matrix Attachment (S/MAR) like DNA element from the gastrointestinal protozoan parasite *Giardia lamblia*

**DOI:** 10.1186/1471-2164-11-386

**Published:** 2010-06-18

**Authors:** Sushma S Padmaja, Jagannathan Lakshmanan, Ravi Gupta, Santanu Banerjee, Pennathur Gautam, Sulagna Banerjee

**Affiliations:** 1 Division of Life Sciences, AU-KBC Research Center, MIT Campus, Chromepet, Chennai 600044, Tamilnadu, India; 2 Center for Biotechnology, Anna University, Sardar Patel Road, Guindy, Chennai 600025, Tamilnadu, India

## Abstract

**Background:**

Chromatin in the nucleus of all eukaryotes is organized into a system of loops and domains. These loops remain fastened at their bases to the fundamental framework of the nucleus, the matrix or the scaffold. The DNA sequences which anchor the bases of the chromatin loops to the matrix are known as Scaffold/Matrix Attachment Regions or S/MARs. Though S/MARs have been studied in yeast and higher eukaryotes and they have been found to be associated with gene organization and regulation of gene expression, they have not been reported in protists like *Giardia*. Several tools have been discovered and formulated to predict S/MARs from a genome of a higher eukaryote which take into account a number of features. However, the lack of a definitive consensus sequence in S/MARs and the randomness of the protozoan genome in general, make it a challenge to predict and identify such sequences from protists.

**Results:**

Here, we have analysed the *Giardia *genome for the probable S/MARs predicted by the available computational tools; and then shown these sequences to be physically associated with the nuclear matrix. Our study also reflects that while no single computational tool is competent to predict such complex elements from protist genomes, a combination of tools followed by experimental verification is the only way to confirm the presence of these elements from these organisms.

**Conclusion:**

This is the first report of S/MAR elements from the protozoan parasite *Giardia lamblia*. This initial work is expected to lay a framework for future studies relating to genome organization as well as gene regulatory elements in this parasite.

## Background

Sequencing and annotation of the different genomes done in the last couple of decades has clearly shown that even the relatively compact eukaryotic genomes have large amounts of non-coding DNA. This DNA harbors elements that control genomic activity such as gene regulators, non-coding RNAs and less well characterized elements that position the chromosomes on the nuclear matrix. The nuclear matrix forms a three dimensional protein network onto which chromatin fibers are attached. Interaction between chromatin and the nuclear matrix is believed to occur at specific sites from 300 base pairs (bp) to several kilobases (kb) long, termed scaffold/matrix attachment regions (S/MAR) [1].

Experimentally, SMARs have been defined as either DNA fragments that remain bound to the nuclear matrix after chromatin proteins and other DNA are removed, or DNA that binds to extracted nuclear matrix in the presence of competitor DNA [2,3]. Identification of S/MARs is a necessary step for successful functional mapping of nucleotide sequences, since these sites can bring genes into association with the nuclear matrix and significantly change transcription level, thus marking transcriptionally active regions [4]. S/MAR elements play a major role in genome organization and gene regulation. They have been reported to alter the expression levels of some genes depending on their position relative to the matrix [5]. S/MARs have also been associated with enhanced transcription, particularly in transgene constructs where flanking transgenes with S/MARs have resulted in higher and more stable expression [6]. They have been associated as a boundary between functional chromatin domains [7,8]. It is also reported that the effects of long-range enhancers may be restricted by the positioning of S/MAR elements [9]. From the genome organization perspective, S/MARs have been implicated in the positioning of chromosomal territories [7,10].

Computational methods are thought to be prerequisite for the analysis of whole genomes for predicting S/MARs and though several tools like MarWiz [11-13], Marscan [8], ChrClass [14], SMARtest [15] and SIDD [16,17] have been developed for this purpose, prediction of S/MAR is not conclusive unless it has been supported by experimental proof. The most common experimental method for identifying S/MAR uses re-association assays to define DNA fragments that bind to the nuclear matrix [18]. South-western assays [19,20] and PCR based assays [21] have also been used successfully to show S/MAR binding to nuclear matrix.

Though S/MARs have been well studied in yeasts, plants, mammalian systems and *Drosophila*, there has been very few reports of these elements from the protists. So far genome wide search for S/MARs have been carried out *in silico *for *Arabidopsis thaliana *and *C. elegans *using SMARTest and MRS finder respectively [22,23]. This study had revealed that genes containing predicted S/MARs had low transcription levels [22]. In *C.elegans*, S/MARs were found to be the flanking coding regions [23]. Marfinder and Marscan have been used previously to identify functional S/MAR elements in *Entamoeba *[19].

The genome of *Giardia lamblia*, the protozoan parasite responsible for causing Giardiasis worldwide among people with poor fecal-oral hygiene, has been sequenced recently [24]. The 11.7 Mb genome of this deep branching eukaryote, distributed over 5 chromosomes showed an exceedingly simple genome structure comprising of only 2 origin recognition complex proteins and total absence of regulatory initiation proteins [24]. Moreover, *Giardia *contained only 4 of the 12 transcription initiation factors present in *Saccharomyces *[25]. As the genome of this organism has been studied, very few regulatory elements were seen to be present in this parasite. Promoters had been identified and characterized earlier [26-32] but other regulatory elements like insulators, boundary elements enhancers and locus control regions were not revealed in the genome sequencing project.

In this work, we have used all the available bioinformatics tools for predicting S/MARs from the genome of *Giardia lamblia *and used PCR based, as well as south-western assays to actually see how many of the predicted S/MARs were able to bind to nuclear matrix. This is the first ever report of S/MAR like DNA elements from this gastrointestinal pathogen. In this paper we have also reflected on how any single computational tools for prediction of S/MAR can be very inaccurate on the protozoan parasite genome, but a combination of different tools along with laboratory based assays, give us a comprehensive idea about S/MAR distribution in *Giardia lamblia *genome. Our studies show 10 S/MAR sequences from *Giardia lamblia *which are associated with its nuclear matrix proteins are can thus be regarded as S/MAR elements.

## Results

### *In silico *prediction of putative S/MARs from *Giardia *genome using existing tools

We used the available tools for identifying putative S/MARs from *Giardia *genome. Currently, four such tools are available: Marfinder [13], Marscan [8], Chrclass [14] and SMARtest [15]. Some groups have also used SIDD (Stress induced DNA Destabilization) for predicting S/MAR regions [16]. However, as SIDD calculations do not [yet] form the basis of an S/MAR predictor for wild type S/MARs in genomic DNA as reflected by Evans et al [33], we have not used it in our study. The results obtained from these tools are summarized in Table 1.

**Table 1:** Summary from various S/MAR identification tools for *Giardia*

Program	# of S/MARs identified	Average length of identified S/MARs (bp)	Av distance between S/MARs (bp)
*SMARTest*	3	375	Too less prediction for calculating loop size.
*Chrclass*	101	554	115,841.58
*MARscan*	218	75	51,364.25
*MAR*finder	66*	735	163,385.3

*Threshold = 20, Window size = 300

**Figure 1:**
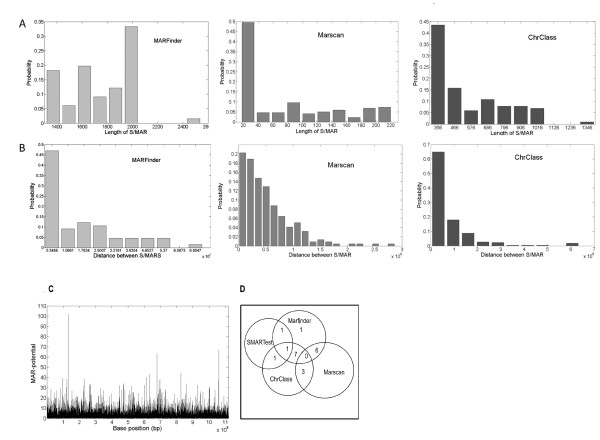
**Analysis of S/MARs from *Giardia *genome using the various available tools like ChrClass, Marfinder and Marscan**. comparing the length of the *Giardia *S/MARs. (A) and distance between two consecutive S/MARs (B). Efficient prediction of S/MAR prediction from *Giardia *genome is achieved by using a combination of two or more tools. Marfinder was by far the best tool for prediction as seen in the profile (C). Marfinder was used in combination with other S/MAR prediction tools for efficient predictions. Venn Diagram (D) shows the number of predictions obtained by the different tools.

SMARtest, which predominantly analyses S/MAR based on the AT richness of the genome gave only 3 hits in the *Giardia *genome. The average percentage of AT for the predicted S/MAR regions using SMARtest is equal to 66.3%. Marscan, which predicts S/MAR regions based on the presence of a bipartite signature, identifies 218 S/MARs from the genome and predicted the average distance between two consecutive S/MARs to be ~50 kb. MARfinder, which is by far the most widely used tool for identifying S/MARs in mammalian and plant genome predicts 66 putative S/MARs in the *Giardia *genome predicting an average distance of ~160 kb between two consecutive S/MARs and Chrclass, the tool based on comparative analysis of various context characteristics associated with S/MARs identifies 100 S/MARs from the *Giardia *genome. A comparison of the results from the different tools in determining the length of S/MARs in the putative S/MAR regions and the distance between S/MARs is demonstrated in Figure 1A Figure 1B and Table 1.

We used Marfinder to understand a complete picture of the S/MAR distribution in the *Giardia *genome (see Figure. 1C). As this method looked into a varied number of features associated with S/MARs (which included topoisomerase binding sites, DNA topology along with AT richness). We trained the software on the negative dataset reported in Evans *et al *[33] prior to running it on *Giardia *genome. It was seen that the threshold value for a "MAR potential" had to be modified from the default values to 50 in order to get a better noise to signal ratio. Using these parameters on the *Giardia *genome, Marfinder predicted 66 putative S/MARs. Evans *et al *[33] has reviewed the existing S/MAR prediction tools and has concluded that no single tool is efficient enough to correctly predict S/MARs even in higher eukaryotes. We thus assumed that in protozoans with a much more "unstructured" and random genome organizations, it would be more prudent to use a combination of tools for the initial prediction of S/MARs. We therefore compared the results from the four different S/MAR predicting programs and selected the S/MAR regions which were predicted by at least two programs one of which was Marfinder (see Table 2). Total of 15 such S/MAR regions were identified. The distribution of S/MARs as predicted by the 4 tools is shown in the Venn diagram in Figure 1D. Primers were designed for all of these 15 putative S/MARs (Additional file 1; Table S1) for further analysis.

**Table 2:** Summary of nuclear matrix binding ability of predicted *Giardia *S/MARs

S/MAR	S/MAR Name	Program	Position		Binding to *Giardia*	nuclear matrix
#			Start	End	By PCR Based Assay	By South Western Assay
1	Glsmar3	MARfinder	608261	609893	Negative	Negative
		MARscan	608909	609128		
2	Glsmar7	MARfinder	1323096	1325120	Negative	Positive
		Chrclass	1323700	1324700		
		SMARtest	1323911	1324345		
3	Glsmar10	MARfinder	2261939	2263576	Positive	Positive
		MARscan	2262911	2262926		
4	Glsmar11	MARfinder	2297402	2299363	Positive	Negative
		MARscan	2298226	2298428		
5	Glsmar16	MARfinder	3366073	3367638	Positive	Negative
		Chrclass	3366600	3366900		
6	Glsmar20	MARfinder	4477903	4479612	Positive	Positive
		MARscan	4478870	4478886		
7	Glsmar66	MARfinder	4479841	4481171	Positive	Positive
		Chrclass	4480200	4480700		
8	Glsmar22	MARfinder	4726045	4728003	Positive	Positive
		Chrclass	4726600	4727300		
9	Glsmar26-1	Chrclass	5228300	5228800	Positive	Positive
		MARfinder	5228326	5230297	Positive	
10	Glsmar26-2					Negative
11	Glsmar39	MARfinder	6968577	6970476	Positive	Negative
		MARscan	6969082	6969238		
12	Glsmar42	MARfinder	7074440,	7076094	Positive	Negative
		Chrclass	7075300	7075800		
13	Glsmar51	MARfinder	9281707	9283667	Negative	Negative
		MARscan	9283630	9283808		
14	Glsmar55	Chrclass	10029900	1003030	Positive	Negative
		MARfinder	10030014	010031712		
15	Glsmar58	MARfinder	10609035	1061158	Negative	Positive
		SMARtest	10610551	110610890		

### Organization of predicted S/MARs in *Giardia*

**Figure 2:**
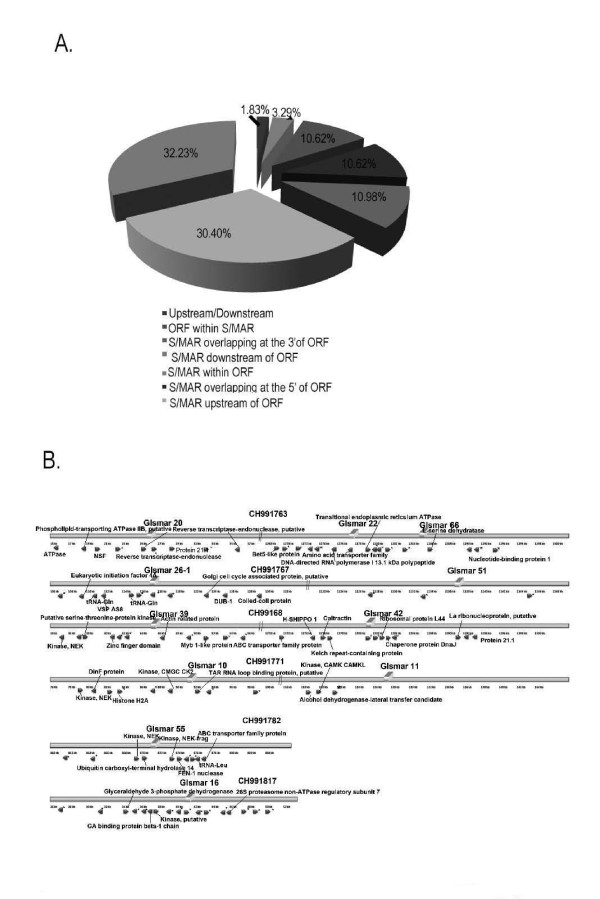
**S/MARs are mostly present in the intergenic regions in the *Giardia *genome**. The pie chart demonstrates the percentage of S/MARs present within ORFs vs number of S/MARs present in the intergenic region (A). Cartoon shows organization of the predicted S/MARs in *Giardia *(B). Bold arrows indicate the ORFs while the solid rectangles represent the S/MARs. S/MARs are numbered and the ORF names are indicated by arrows.

Of all the S/MARs predicted from the *Giardia *genome, our analysis shows that about 30% of the S/MARs are present in the upstream or downstream of ORFs and thus in the intergenic region. 10% S/MARs overlapped with ORFs either in the 5' or 3' region of the gene. Also, about 10% of S/MARs contained ORFs within themselves and 3% S/MARs were present within the ORFs. This distribution of S/MARs with respect to ORFs is shown in Figure 2A.

Of the 15 putative S/MARs, some were found to have some interesting organization. GlSMAR7 (Figure 2B) was found to have ORF of a reverse transcriptase endonuclease apart from a VSP and High Cysteine protein within 12 kb of it. Similar organization i.e presence of several reverse transcriptase endonuclease was also noticed in GlSMAR22 (Figure 2B) These ORFs are reported to be present in the telomeric region of the chromosome in *Giardia *[34]. The significance of the presence of these elements in close proximity of such ORFs is beyond the scope of current study. The 15 putative S/MARs were then tested experimentally for their ability to bind to nuclear matrix. The results are summarized in Table 2.

### *Giardia *S/MAR like elements are present in the nuclear matrix

**Figure 3:**
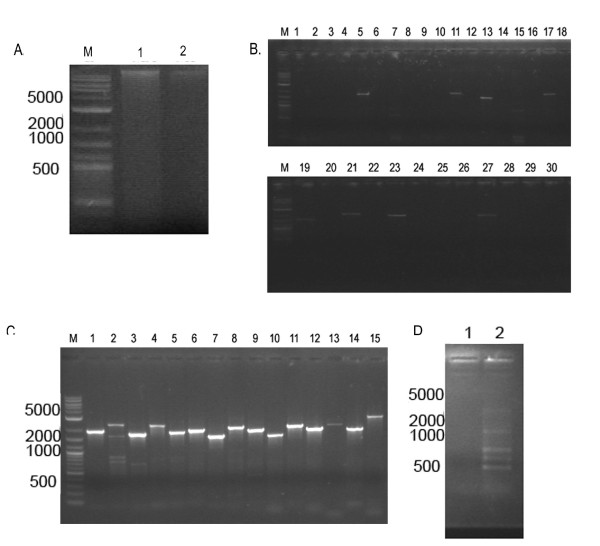
**PCR based assay of nuclear matrix associated DNA from *Giardia***. Nuclear matrix dependent (A;Lane 1) and independent DNA (A;Lane 2) was extracted from nuclear matrix (see methods) according to [20 and used for PCR reactions with predicted S/MAR primers(B). Lane M is the DNA marker (NEB); lanes 1-30 are PCR products of which odd number lanes (1,3,5,7.....27,29) even numberedare nuclear lanes (2,4,6...26,28,30) are nuclear matrix from the *Giardia *genomic DNA as a positive control(C). The PCRs are loaded in the following order: GlSMAR3, GlSMAR7, GlSMAR10, GlSMAR11, GlSMAR16, GlSMAR20, GlSMAR66, GlSMAR20, GlSMAR22, GlSMAR26-1, GlSMAR26-2, GlSMAR39, GlSMAR42, GlSMAR51, GlSMAR55, GlSMAR58, in both panel B and C. A non-S/MAR sequence was used as a control (PanelD). Amplification of multiple bands was seen in the matrix independent fraction (loop-fraction) lane 2 and no amplification was seen in the matrix dependent fraction.

To determine if *Giardia *S/MARs predicted by the bioinformatics search were indeed present in the nuclear matrix, we performed a PCR based test [21]. The extracted DNA from the nuclear matrix as well as the supernatant released after *Eco*RI digestion (Figure 3A) were used for polymerase chain amplification with the primers (additional file 1, Table S1). It was seen that GlSMAR10, GlSMAR11, GlSMAR20, GlSMAR66, GlSMAR22, GlSMAR26-1, GlSMAR26-2, GlSMAR39, GlSMAR42 and GlSMAR55 amplified at the expected size (Figure 3B), whereas, no amplicon was seen from the supernatant fraction. As positive control, these fragments were also amplified from *Giardia *genomic DNA (Figure 3C). A non S/MAR DNA sequence was used as a negative control (Figure 3D). This fragment was seen to be amplified from the loop fraction (Figure 3D Lane 1) and not present in the attached fraction. (Figure 3D Lane 2). Thus, at least 10 of the 15 *Giardia *S/MAR sequences predicted by the different computational tools were associated with *Giardia *nuclear matrix (Figure 3A). GlSMAR58 and GlSMAR16 did not amplify from this DNA pool as they had *Eco*RI sites internal to the primer binding sites. It was observed that GlSMAR 20 also had *Eco*RI sites in the sequence. However, GlSMAR20 had its MAR potential peaks coinciding with this positions. It is therefore possible that the *Eco*RI sites in GlSMAR20 are protected from digestion as this region is actively involved in binding to the proteins in the nuclear matrix. However, this can only be confirmed by further experimentation. It may be noted that though the length of S/MARs in other eukaryotes range from 300-700 bp, the *Giardia *S/MARs were found to be longer. Fragments of 1.8-2.3 kb range were amplified from the matrix associated DNA pool. The exact length of DNA which is essential for the binding remains to be determined by further experimentation.

### *Giardia *S/MARs bind to nuclear matrix proteins

**Figure 4:**
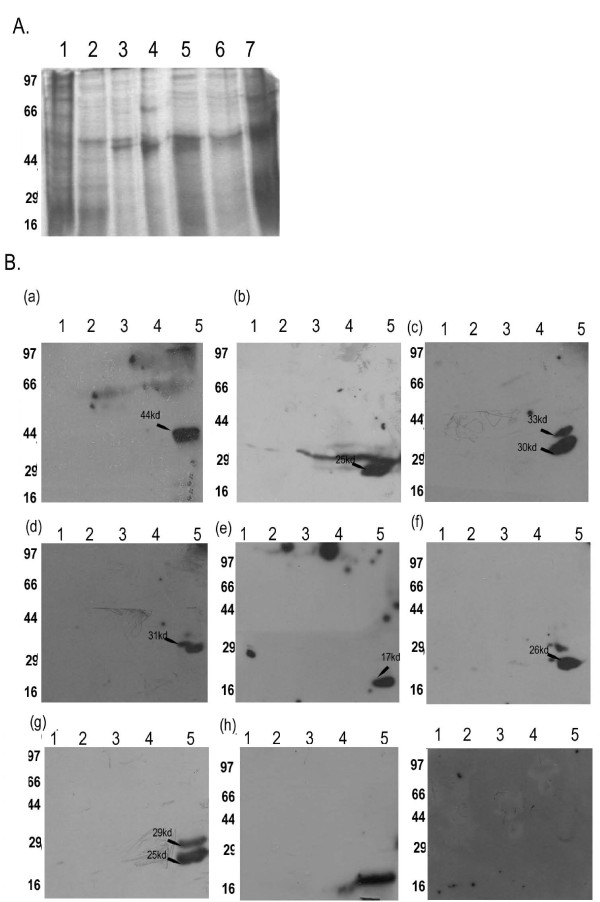
**South western assay with Giardia S/MARs**. Nuclear Matrix was isolated from *Giardia *cells and the different fractions were separated on a SDS-PAGE (Panel A lane 6). The other fractions are cytoplasm (lane 1)Extract after DNAse treatment (lane 2); extract after treatment with 50 mM NaCl (Lane3) and extract after treatment with 1.6 M salt (Lane 4 and 5). Panel B shows the southwestern hybridization of the nuclear farctions of *Giardia *separated in a SDS-PAGE, with the different probes. 8 of the 14 predicted *Giardia *S/MARs are able to bind to nuclear matrix. (Lane5; 4B-I) in all the panels. Only GlSMAR10 (4b lane 4)) shows binding to 1.6 M salt extract along with nuclear matrix proteins. All others GlSMAR7 (panel a); GlSMAR16 (panel c); GlSMAR20 (panel d); GlSMAR22 (panel e); GlSMAR26 (panel f) GlSMAR58 (panel g) and GlSMAR66 (panel h) bind only to nuclear matrix proteins. Panel I is a negative control probed with linear digest of pUC19 DNA, which shows no binding to nuclear matrix or high salt extract.

Next, we tested the nuclear matrix associated putative S/MAR sequences for their nuclear matrix binding by chemiluminiscence. Common S/MARs predicted by the bioinformatic tools were developed as probes. These S/MARs were tested for their ability to bind to nuclear matrix isolated from the organism. Nuclear matrix was isolated according to the protocol of Kauffmann et al, 1991[35]. In this method, isolated nuclei were first treated with DNAse to digest the DNA that was not associated with any proteins. The resulting pellet was then extracted with 1.6 M NaCl to release the histones and other high salt soluble proteins. This was followed up with a detergent wash. The residual pellet, inextractible by detergent or high salt was the nuclear matrix that was used for the different experiments. Binding to the nuclear matrix fraction was done by south western blotting [20]. It was seen that 8 of the 15 predicted S/MARs were able to bind to nuclear matrix proteins (GlSMAR7, GlSMAR10, GlSMAR16, GlSMAR20, GlSMAR22, GlSMAR26-1, GlSMAR58 and GlSMAR66). All these 8 S/MARs were also seen to be associated with the nuclear matrix in the PCR based assay. 4 of the S/MARs which were seen to be associated with the nuclear matrix in the PCR based assay did not show binding to nuclear matrix in the south western assay (GlSMAR11, GlSMAR39, GlSMAR42, GlSMAR55). This was probably because the DNA binding motifs in the nuclear matrix were not exposed to the probes in these cases. Alternately, GlSMAR58 and GlSMAR16 showed binding to the nuclear matrix by southwestern though it was not amplified from the matrix dependent pool in the PCR based assay. This was because this S/MARs had *Eco *RI sites internal to the primers that were used for amplifying the S/MARs from the matrix dependent fraction. GlSMAR7 did not amplify in the PCR based assay, but it showed binding to the nuclear matrix proteins in the south western assay. We checked the sequence for the presence of EcoRI sites but could not find any. It is possible that the template for the GlSMAR7 was too low in the matrix dependent DNA pool to be amplified by the GlSMAR7 primers. When the probe was prepared for the south western assay, it was thus able to bind to the protein in the nuclear matrix. All S/MARs recognized proteins in the *Giardia *nuclear matrix fraction ranging a molecular weight of 17 kd-44 kd (Figure 4B a-g).

### Mass Spectrometry of *Giardia *nuclear matrix protein

To verify whether any of the proteins recognized by the S/MARs from *Giardia *were indeed resident nuclear matrix proteins, we excised one band (44 kd) from the coomassie stained gel which also bound to GlSMAR7 (Figure 4B a) and went for mass spectrometric identification.

**Figure 5:**
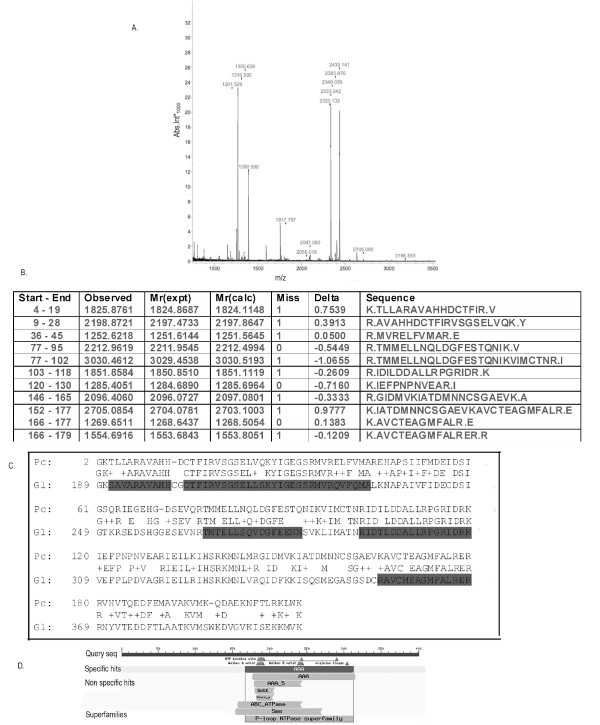
**MALDI-TOF analysis of the *Giardia *nuclear matrix protein**. A ~42 kd protein excised from the coomassie stained gel and analysed by MALDI-TOF, showed distinct and sharp peptide profile in the mass spectrometric analysis (Panel A). The peptides obtained (panel B) were aligned with the *Giardia *protein giving the closest hit (panel C). This protein, a proteasome 26 S subunit 8 had a conserved domain of AAA or **A**TPAse **A**ssociated with diverse cellular Activities (Panel D).

The obtained peaklist (Figure 5A) was analysed using the web based analysis software MASCOT against the NCBInr database as *G.lamblia *genomic database is not present in MASCOT. The top score was that of a homologue of a tat-binding protein from *Plasmodium chabaudi*((M*r *= ~20 kDa). The peptides identified in the peaklist by MASCOT are shown in Figure 5B. When searched against the *Giardia *database with the *P. chabaudi *protein sequence, as well as the individual peptides from the MS data, a 26 S protease regulatory subunit 8 in *G.lamblia *showed 63% identity with 79% homology (M*r *= 44 kDa) (Figure 5C). This protein complex is a known resident nuclear matrix protein in higher eukaryotes [36] and in *G.lamblia*, this subunit contains an AAA domain of ATPase associated with diverse cellular activities. This class of proteins belongs to the superfamily of ring shaped P-loop ATPase [Figure. 5D]. These proteins exert their activity through energy dependent unfolding of macromolecules and are also reported to be involved in regulation of gene expression [37,38].

## Discussion

*Giardia lamblia*, is a diplomonad, with 2 nuclei and is often referred to as a "deep branching eukaryote" as it diverged out of the main evolutionary tree long before the other eukaryotes. As a result, this oraganism has a number of unique features which have become more "organized" in the higher eukaryotes. One of the most unique features of *Giardia *is its lack of organellar structures as for example a well defined mitochondria, Golgi bodies and endoplasmic reticulum, in spite of being an eukaryote. Traces of marker proteins from these organelles and an amazingly developed membrane structure adept to carry out these functions are however present here enabling this organism to be classified as a eukaryote [39].

Though the initials reports of nuclear matrix go as far back as 1960's, the research on S/MARs as potential regulatory elements come from the works of J. Bode in 1988 [40,41]. Since then, throughout the eukaryotic world, the S/MARs have been found to play a significant role in the organization of chromatin, and gene regulation. Studies on the recently sequenced *Giardia *genome have shown the genome to be unique in its own way. The protist has 5 chromosomes, and almost 9000 ORFs packed into a small genome of 12 Mb length. It has been seen that the parasite has no homolog for H1 which is the universal linker for compacting chromatin in the nuclei [26]. In this scenario, the study of Scaffold/Matrix attachment region in this parasite can shed adequate light on the chromatin organization in this organism. We did a preliminary screen on the *G.lamblia *genome with available S/MAR prediction tools. When the common regions predicted by at least 2 tools were taken into consideration, we were able to shortlist at least 15 putative S/MAR regions. To prove this DNA fragments were indeed nuclear matrix dependent fragments we did the PCR based assay, which showed that 10 out of 15 putative S/MARs where actually associated with the nuclear matrix of *Giardia*. This showed that the false positive rate of our strategy was about 33%. Assuming that the distance between the S/MARs in this genome can range from 50-160 kb, as seen in Table 1, we expect about 110 S/MARs in the entire genome of *Giardia*. The combination of computational tools correctly predicted only 10% of the total number of the expected S/MARs. This indicates that the S/MAR prediction tools that can be used with accuracy on the higher eukaryotic genome, in most of the instances are not very accurate in predicting lower eukaryotic S/MARs. Experimental methods are an absolute necessity in correctly identifying these elements from the lower eukaryotic genomes. The computational tools for S/MAR predictions can only be used as an initial screen for scanning the genome of the protists for presence or absence of S/MARs, but the actual confirmation is achieved only by experimental methods. Of the 10 S/MARs, 8 also showed positive nuclear matrix binding property in south western blots. Among these, 7 S/MARs which showed positive binding both in PCR as well as south western assay were indeed true S/MARs. It now remains to test these *Giardia *S/MARs for chromosomal organization studies.

One of the major properties of S/MARs is chromosome organization, anchoring and maintenance of higher order structure [42]. This is achieved by the proteins in the nuclear matrix which bind to the S/MARs thereby allowing it to carry out these functions. The proteins in the nuclear matrix are involved in a host of different functions, including DNA replication and repair [43]. Of these the S/MAR binding proteins are (S/MARBP) are of utmost importance as they regulate transcription, replication, repair and regulation of gene expression [44]. One of the GlSMARs, GlSMAR7 bound to a proteasome subunit 8 as shown by our mass spectrometry results. The 26 S proteasome is an eukaryotic ATP-dependent protease complex of 2000 kd which is reported to be present in the nuclear matrix in mouse myoblasts [36]. As seen in Figure 5, the conserved domain in the 26 S proteasome subunit 8 in *Giardia *was a AAA domain belonging to the ATPase binding protein superfamily. These proteins perform a diverse range of functions in the cell starting vesicle fusion, peroxidase biogenesis [45] to DNA repair [46]. Thus it is not unlikely that this protein would be associated with S/MARs and have DNA binding properties. There have been reports on the proteasome 20 S of *Giardia lamblia *[47,48], where Emmerlich *et al *showed the 14 subunits making up this proteasome structure. Though the annotated genome of *Giardia *shows the presence of several of the proteasome 26 S subunits, no detailed analysis has been done on these proteins in *Giardia*. A detailed phylogenetic analysis of another AAA ATPase domain containing protein Midasin has been studied by Gallego *et al*. [49]. This protein is conserved thoughout eukaryotes and plays the role of a nuclear chaperone in most organisms. One of the proteins found to be associated with S/MARs from yeast to humans, is the SAF Box domain containing protein. As reported by Kipp et al in 2000 [50], SAF-A binds to S/MARs through a novel conserved protein domain. A search in http://www.eupathDB.org for proteins having the SAF box or the SAP domain showed that 47 such proteins were present in the different protozoan genomes (*Cryptosporidium, Plasmodium, Toxoplasma, Entamoeba *and *Trichomonas*). Thus it is likely that these genomes will also have S/MAR like elements in their genome. However, when searched in the *Giardia *genome, this SAF/SAP domain containing protein was not present. Our experimental results discussed in this work indicate that *Giardia *has S/MAR binding protein (26 S proteasome subunit 8) which does not have a SAF/SAP domain, but has nucleotide binding domains. While it is possible that in a recently sequenced genome, this protein was not annotated, it is also possible that *Giardia *placed much earlier in the evolutionary scale probably has not yet defined a machinery where these highly conserved domain containing proteins may be present. The presence of S/MARs in *Giardia *and the absence of SAF box proteins in this organism may also indicate that the early divergence of *Giardia *during evolution probably .resulted in "missing out" this very conserved protein involved in nuclear architecture.

S/MARs have been found to be associated with not only chromatin anchoring but also with other regions of the genome as introns [51] and can play a significant role in the regulation of gene expression [52,53]. Studies on S/MAR in *Arabidopsis *and maize [54,55] have shown that the plant genome is not packaged by random gathering into domains of indiscriminate length, but rather, the genome is gathered into specific domains, and a gene consistently occupies a discrete physical section of the genome. The average loop size in *Arabidopsis *and maize has been estimated as 25 and 45 kb, respectively [55], though other studies [8] have suggested smaller domain sizes. Some loops may remain permanently condensed and inactive, even within the euchromatic portions of the genome, whereas others can be extended to produce a transcriptionally poised conformation in appropriately differentiated cells [56]. Our analysis for a genome-wide distribution of S/MARs using different tools indicates that the loop size ranges from 50-160 kbp in *Giardia *(Table 1). Data on the location of transcribed elements within structural loops at the supragenic level suggest that attachment to the matrix and transcription is not systematically associated [57,58], though S/MARs are associated with the ends of some DNaseI-sensitive (transcriptionally poised) domains [59]. S/MARs have also been identified within introns of genes [60,61]. Cockerill et al. [60] suggested that S/MARs flanking enhancer sequences may act as positive and/or negative regulators of enhancer function. It is presumed that additional specific S/MARs have been further demonstrated in a variety of functional tests to act as insulators [61], according to the loop domain model, by protecting a loop from the effects of the neighboring chromatin or associated enhancer sequences. Distribution of *Giardia *S/MARs among the transcription factors also hints at this possibility (data not shown). A much more in-depth study of the S/MARs in lower eukaryotes is required to understand the chromatin dynamics and packing in these organisms.

An observation was made in the study by Linnemann *et al *in 2009[62], where it was seen that the S/MARs when present in the 5' region of a gene resulted in a transcript presence, where as those present within the ORF associated with silenced genes. A number of S/MARs in *Giardia *were also found within the ORFs. The significance of this is not clear. In *Entamoeba*, such S/MARs were found to have reduced binding ability to nuclear matrix compared to the ones that were present outside ORF (our unpublished data). It is possible that in these early eukaryotes, the genome organization machinery is also in early stages of evolution and the S/MARs within the ORFs are actually the ones which in course of evolution would lose their ability to bind to the nuclear matrix completely.

## Conclusions

Though analysis of S/MAR on large genomic sequences are being done [63-65], S/MAR regions of protists have never really come to the limelight. Study on the S/MARs in these organisms is of significance in the understanding their gene organization and regulation. The multiple roles played by these S/MARs starting from chromosome organization to promoter control, acting as domain barriers, make them important regulatory elements which have not received much focus yet. Most of the prediction tools are designed with the structurally organized higher eukaryotic genome in mind. A comparison of these tools reveal that no single tool is accurate enough to predict the S/MARs even from an organism with a well defined genome structure [33]. In case of lower eukaryotes, these tools do identify the S/MARs, but with much less accuracy. Our study clearly indicated that even if we take into consideration all the available tools for predicting S/MARs from protozoan parasites, they have to be verified experimentally for their ability to be associated with the nuclear matrix. Studies like this, also indicate the need to modify and develop more dedicated tools for the prediction of these elements from such divergent genomes, which in turn would help to study gene organization and gene regulation in a much wider scale in these protozoans.

## Methods

### Bioinformatic tools for prediction of S/MARs

*G. lamblia *genome sequences available at http://www.Giardiadb.org were used for all analysis. S/MAR analysis was done according to the available S/MAR analysis tools - Marfinder (downloaded from http://genomecluster.secs.oakland.edu/marwiz/) SMARTEST (http://www.genomatix.de/smartest.html), Marscan (EMBOSS) and Chrclass (http://ftp.bionet.nsc.ru/pub/biology/chrclass/chrclas2.zip). The tools and the description of the datasets as well as the parameter information were done according to Evans *et al *[25], with modifications wherever required.

### *Giardia *cell culture

*Giardia lamblia *strain WB1267 was cultured axenically in TYI-S media supplemented with 10% Adult Bovine serum (Invitrogen) and 1 mg/ml of bovine bile (Sigma) in 50 ml culture flasks. Parasites were routinely subcultured every 48-72 hours when confluent. Cells were harvested for nuclear matrix isolation by chilling on ice for 20 mins followed by harvesting at 2000 rpm for 5 min in extraction buffer (10 mM Hepes, pH 6.8; 24 mM KCl; 10 mM MgCl_2_) in the presence of protease inhibitor cocktail (Sigma, USA).

### Genomic DNA, Designing primers and PCR

*Giardia lamblia *(strain WB1267) genomic DNA was prepared from confluent *Giardia *cultures using the Genomic DNA Isolation kit (Sigma) according to the manufacturers instruction. Primers were designed against the predicted S/MAR sequences (details in additional file 1; Table S1) and supplied by Ocimum Biosolutions, India. Putative S/MARs were amplified from the *G. lamblia *genome by Polymerase Chain Reaction using Taq polymerase (NEB, USA).2 ng G.lamblia genomic DNA was used as a template. DNA was denatured at 95°C for 5 min, followed by 30 cycles of denaturation at 95°C for 30 s, annealing at 55°C for 45 s and extension at 72°Cfor 30 s. Final extension of 72°C was kept for 7 min. Amplicons were sequenced to confirm the genomic sequence.

### Synthesis of probes for hybridization

The purified PCR products were used as templates for the labeling reaction. Biotinylated dNTPs (NEB) and NEBlot Kit (NEB) was used to label the probes for chemiluminiscent detection. The reaction for synthesis of probes was done according to the manufacturer's instruction.

### Isolation of nuclear matrix

1. Cultured *G. lamblia *cells were harvested at 2000 rpm and washed once in phosphate buffered saline (PBS). The cells were lysed in Extraction buffer (10 mM HEPES-KOH(pH 7.2), 24 mM KCl, 10 mM MgCl, 1 mM E64 (trans-epoxysuccinyl-L-leucylamido-(4-guanidino)butane), a protease inhibitor; 1 mM, PMSF, 2 mM DTT, 0.03%NP-40). The lysate was loaded on a cushion of Extraction buffer containing 0.8 M sucrose and centrifuged at 6000 rpm for 20 minutes. The nuclei were recovered in the pellet.

Nuclear matrices were prepared by treatment of the isolated nuclei with 50 U of DNase I at 37°C for 30 minutes and centrifuged at 6000 rpm for 10 minutes [18]. The pellet was then washed twice with Low Salt Buffer (LSB) containing 10 mM HEPES-KOH(pH 7.2), 0.2 mM MgCl2, 10 mM 2-mercaptoethanol followed by treatment with High Salt Buffer (HSB) containing 1.6 M NaCl, 10 mM HEPES-KOH(pH 7.2), 0.2 mM MgCl2,10 mM 2-mercaptoethanol and incubated at 4°C for 15 minutes. The insoluble nuclear matrix proteins were separated from the high salt extractable proteins by centrifugation at 6000 rpm for 10 minutes. The final pellet was further washed with 0.5% Triton-X 100. All fractions were prepared in SDS-PAGE gel loading buffer and separated on a 10% SDS-PAGE.

### PCR-based assay for S/MAR binding

Nuclear matrix was isolated from *G.lamblia *cells as described above. For the PCR based assay the protocol of Kramer [20] was used with modifications. Briefly, *Giardia *nuclei were washed once with LSB and then treated with HSB and incubated on ice for 15 min. Following incubation, the reaction mix was centrifuged at 6000 rpm and the supernatant was removed. The pellet was washed once again with LSB followed by 1 × restriction enzyme buffer for *Eco*R1. The pellet was then digested with *Eco*R1 for 2 hrs at 37°C. Following digestion, the reaction tube was centrifuged at 6000 rpm for 10 min and the supernatant was collected in a fresh tube. The residual pellet (nuclear matrix) and the supernatant were subjected to phenol: chloroform (1:1; v/v) treatment and the extracted DNA from both fractions were precipitated with equal volume of isopropanol. 2 ng of the extracted DNA was next used as a template for PCR for the different predicted S/MAR sequences in *Giardia*.

### South western hybridization for detecting nuclear matrix-S/MAR association

The protein fractions separated by SDS-PAGE were transferred to PVDF membrane. The membrane was blocked with 3% non-fat skimmed milk in containing 20 mM Tris-HCl (pH 8.0), 50 mM NaCl, 1 mM EDTA (Standard Binding Buffer; SBB) for 2 hours. After three washes of 15 minutes with SBB the membrane was incubated overnight at 4°C with the biotinylated DNA probes. Unbound probe was washed with SBB followed by incubation with Streptavidin conjugated to Horseradish Peroxidase (Sigma 1:500 dilution) for 1 hour. Excess Streptavidin -HRP was washed with the same buffer. The DNA binding ability was then detected with an enzyme catalyzed light emitting reaction using Super Signal West Pico Chemiluminescent substrate kit (Pierce 34082) according to the manufacturer's instruction. The membrane was then exposed to CL-Xposure films (Pierce 34092) and the emitted light was captured on the film.

### Sample preparation for proteomic analysis of *Giardia *nuclear matrix protein

A major band around 44 kDa was excised from the gel and sent to Syngene International, Bangalore, India for proteomic analysis.

The sample processing was done by the CRO according to standard methods. Briefly, the gel bands supplied were washed with water and chopped into ~1 mm cubes and washed with 50 mM NH4HCO3 and acetonitrile mixture (1:1) for 15 min and washing solution was aspirated completely. Sufficient acetonitrile was added to cover the gel particles following above the washing step. Acetonitrile was removed after 2 min and gel pieces were re-hydrated in 50 mM NH4HCO3. After 5 min, an equal volume of acetonitrile was added and incubated for 15 min followed by complete removal of all solvents. Gel pieces were covered by enough acetonitrile to effect shrinking of gel pieces. Following shrinkage of gel pieces, acetonitrile was removed and gel particles were dried in a vacuum centrifuge. For reduction and alkylation, the gel particles were allowed to swell in 50 mM NH_4_HCO_3_, 10 mM dithiothreitol (DTT) and incubated for 45 min at 56°C in a water bath followed by cooling to room temperature. Excess liquid was removed and replaced with freshly prepared 50 mM iodoacetamide in 50 mM NH4HCO_3 _followed by incubation for 30 min at room temperature in the dark. Excess iodoacetamide solution was removed and gel particles were washed twice with 50 mM NH_4_HCO_3 _and acetonitrile mixture (1:1). Each washing was carried out for 15 min. Gel pieces were allowed to dehydrate in acetonitrile followed by vacuum drying. For in-gel digestion, gel pieces were rehydrated in 20 ng/ul Trypsin (Sigma) solution prepared in 25 mM NH_4_HCO_3 _at 37°C for 30 min.25 mM NH_4_HCO_3 _was added to the reaction mixture so that the gels remained completely submerged. Digestion was allowed to proceed at 37°C for 16 h.

Following digestion, the peptides were extracted by adding extraction buffer (50% acetonitrile containing 0.1% TFA) to cover the gel pieces followed by sonication. The extract was collected after centrifugation and concentrated to a final volume of 10μl using vacuum centrifuge.

### MALDI matrix preparation and MALDI-MS Analysis

Saturated solution of Alpha-cayano-4-hydroxy cinnamic acid was prepared using 30% acetonitrile containing 0.1%TFA to prepare the matrix for MALDI. Undissolved matrix particles were removed by centrifugation. Equal amount of sample and matrix were mixed in a microfuge tube and spotted on MALDI-target plate and the mixture was allowed to dry at room temperature.

MALDI spectra were acquired in an AUTOFLEX III SMARTBEAM MALDI-MS instrument (Bruker Daltonics, Germany). External calibration was done with peptide calibration standard supplied by Bruker, with masses ranging from 1046 Da-3147 Da. All the spectra were acquired in Reflectron +ve ion mode with an average of 2000 laser shots. Mass detection range was set between m/z 800-3500. Acquisition software used was FlexControl version 3 and Analysis software used was FlexAnlaysis version3. Analysis of the peaklist obtained was done using the web based analysis software MASCOT using the NCBInr database. All the default parameters of MASCOT were maintained for analysis.

## Abbreviations

PCR: Polymerase Chain Reaction; S/MAR: Scaffold/Matrix Attachment Regions.

## Authors' contributions

SP performed the PCR, southwestern assays and PCR based assays; JL and RG analysed the genome sequences for the S/MAR sequences using the different tools and shortlisted the different S/MARs to be tested and performed statistical analysis wherever required;, Santanu Banerjee and Sulagna Banerjee prepared samples for proteomics studies and analysed the proteomics data; PG,, Santanu Banerjee and Sulagna Banerjee designed experiments and coordinated the study; Sulagna Banerjee analysed data, and prepared manuscript. All authors read and analyzed the final manuscript.

## Supplementary Material

Additional file 1 **Table S1**. List of forward and reverse primers for amplifying predicted S/MARs from *Giardia*. Click here for file
