# Supplementary material for: Identification of scaffold/Matrix Attachment (S/MAR) like DNA element from the gastrointestinal protozoan parasite Giardia lamblia
Source: BMC Genomics. 2010 Jun 18;11:386. doi: 10.1186/1471-2164-11-386 (PMC3017767; doi:10.1186/1471-2164-11-386)
Supplement: Additional file 1 — Table S1. List of forward and reverse primers for amplifying predicted S/MARs from Giardia. [file 1471-2164-11-386-S1.DOCX]

Additional Table;

Table S1: List of forward and reverse primers for amplifying predicted S/MARs from *Giardia*

| Name | Forward primer | Reverse primer |
| --- | --- | --- |
| Glsmar3 | **T G G C C G A G T T T G T T A T T G C A** | **G T T C C A A C G G T C G T C T T C A T** |
| Glsmar7 | **G C T C T T C T T C G C C A G G T C T A** | **T G G T G A G G A G G T C T C T T G C T** |
| Glsmar10 | **C T T C A G A G T C C T T C G G T T G C** | **A T A T T G G A G C A G G C G C A T T A** |
| Glsmar11 | **G G A G T A A C T C G C G G T T G A A G** | **T G A T G C T C C G T A C T T T G C A C** |
| Glsmar16 | **T A A C A G C A G C A A C A G C A A G G** | **G C C C T C T T G C C T C T C C T A G A** |
| Glsmar20 | **A C T T C G A A T A C G G C T C T G G A** | **G T C T G G C C A A T C T G T G G A A T** |
| Glsmar22 | **G T C A A T A G T T C G G G C A T C G T** | **T G G T G A G A G A C G A C T C G T T G** |
| Glsmar26 | **A C T G A C A T G A C G C A G A C G T T** | **G T C T T C C A G A G T C C G A A T G C** |
| Glsmar39 | **A C T G G T T T T C C T T C G C A A T G** | **G C A A A T T C G C T T G G G A T A A G** |
| Glsmar42 | **C C G A T T G G A C A G G A A G T C T C** | **C G A G A G C T A C G C C T A T T T G G** |
| Glsmar51 | **C C A A A A C G C C T T C T G A T C T C** | **T T G C A C A C A G C A A C G T A C A G** |
| Glsmar55 | **G A G G A C G A C C C T G A C A G A A C** | **G T A G C A C A A A G G C C A C C A A T** |
| Glsmar58 | **A C A C A A T G C A G A T G G T C T C G** | **T T T C A G T C G C A T G A G T C C A G** |
| Glsmar66 | **C T T A C C G A C A A G G C A A T G C T** | **C G C T C G G A G G A C T C A G A A T A** |
